# Regulation of the mPTP by SIRT3-mediated deacetylation of CypD at lysine 166 suppresses age-related cardiac hypertrophy

**DOI:** 10.18632/aging.100252

**Published:** 2010-12-29

**Authors:** Angela V. Hafner, Jing Dai, Ana P. Gomes, Chun-Yang Xiao, Carlos M. Palmeira, Anthony Rosenzweig, David A. Sinclair

**Affiliations:** ^1^ Harvard Medical School, Department of Pathology and Glenn Labs for Aging Research, Boston, MA 02115, USA; ^2^ Freie University Berlin, Institute for Chemistry and Biochemistry, Berlin, 14195 Germany; ^3^ University of Coimbra, Center for Neurosciences and Cell Biology, 3004-517 Coimbra, Portugal; ^4^ Beth Israel Deaconess Medical Center, Cardiovascular Division, Harvard Medical School, Center for Life Science, Boston, MA 02115, USA

**Keywords:** sirtuin, SIRT3, mitochondria, acetylated Cyclophilin D, cyclosporine A, acetylation, cardiac, heart, mitochondrial permeability transition, mPTP

## Abstract

Cardiac failure is a leading cause of age-related death, though its root cause remains unknown. Mounting evidence implicates a decline in mitochondrial function due to increased opening of the mitochondrial permeability transition pore (mPTP). Here we report that the NAD^+^-dependent deacetylase SIRT3 deacetylates the regulatory component of the mPTP, cyclophilin D (CypD) on lysine 166, adjacent to the binding site of cyclosporine A, a CypD inhibitor. Cardiac myocytes from mice lacking SIRT3 exhibit an age-dependent increase in mitochondrial swelling due to increased mPTP opening, a phenotype that is rescued by cyclosporine A. SIRT3 knockout mice show accelerated signs of aging in the heart including cardiac hypertrophy and fibrosis at 13 months of age. SIRT3 knockout mice are also hypersensitive to heart stress induced by transverse aortic constriction (TAC), as evidenced by cardiac hypertrophy, fibrosis, and increased mortality. Together, these data show for the first time that SIRT3 activity is necessary to prevent mitochondrial dysfunction and cardiac hypertrophy during aging and shed light on new pharmacological approaches to delaying aging and treating diseases in cardiac muscle and possibly other post-mitotic tissues.

## INTRODUCTION

The “Mitochondrial Theory of Aging” posits that a decline in mitochondrial function over time is an underlying cause of aging [1, 2]. There are many observations consistent with this theory, including that mitochondria of young animals are relatively small and bioenergetically efficient but over time they become less numerous, swollen, and chronically depolarized [3].

Increasing evidence implicates a multi-protein complex called the mitochondrial permeability transition pore (mPTP) in the decline in mitochondrial dysfunction with age. Within young cells, mitochondria are impermeable to most ions and solutes, allowing them to maintain membrane potential and store Ca^2+^[4]. As age advances, however, small disturbances to cellular homeostasis increasingly trigger mPTP formation. Well known triggers include increased levels of ROS and increased Ca^2+^[5]. Acute triggering of the mPTP can lead to apoptosis whereas low-level, chronic triggering results in mitochondrial swelling, membrane de-polarization, and the destruction of defective mitochondria by autophagy [6]. After three decades of research, the mechanisms that underlie increased mitochondrial membrane permeability with age remains poorly understood.

The structure of the mPTP includes the voltage-dependent ion channel (VDAC), adenine nucleotide translocator (ANT), and cyclophilin D (CypD). Binding of CypD to ANT initiates a tunnel-like structure that connects the mitochondrial matrix with the cytosol [7]. The therapeutic value of maintaining the mPTP in a closed state is highlighted by the resistance of CypD knockout mice (Ppif^−/−^) to a variety of diseases, including autoimmune encephalomyelitis (EAE), sarcoglycan deficiency, laminin deficiency, collagen VI deficiency, ALS (SOD^G93A^), cardiac ischemia-reperfusion injury, and Alzheimer's disease (AD) [8, 9]. These findings have made CypD a drug target of high interest [10, 11]. Unfortunately, pharmacological inhibitors of CypD, such as cyclosporine A (CsA), also inhibit the structurally-related cyclophilins CypA and CypB, which are critical for immune function [12]. Thus, understanding how the mPTP is regulated could identify ways to specifically inhibit the mPTP without compromising immunity.

The mammalian sirtuins are a family of seven protein deacetylases that are increasingly thought to underlie many of the health benefits of calorie restriction [13]. What makes sirtuins distinct from other deacetylases is their requirement for NAD^+^ as a co-substrate [14]. There are three mitochondrial sirtuins, SIRT3, SIRT4 and SIRT5, which are known to regulate fatty acid oxidation, respiration, amino acid metabolism, and the urea cycle [15, 16]. Known substrate targets include acetyl coenzyme A synthetase 2 (ACS2) [17, 18], glutamate dehydrogenase (GDH) [19], and long-chain acyl coenzyme A dehydrogenase (LCAD) [20]. SIRT3 can also act as a tumor suppressor by lowering levels of ROS and possibly by altering cellular metabolism [21].

**Figure 1.**
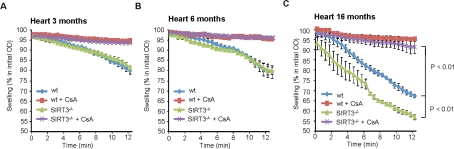
SIRT3 prevents mitochondrial permeability transition in cardiac tissue during aging. Mitochondrial swelling induced by Ca^2+^. Mitochondria from 3 (**A**), 6 (**B**) and 16 months old (**C**) wt and SIRT3^−/−^ mouse hearts subjected to Ca^2+^-induced mitochondrial swelling, measured as % decrease in the initial optical density (OD540) in the presence or absence of 1 μM CsA (n=4 per group and age). All error bars show s.e.m.

We recently reported that cell survival in response to genotoxic stress is governed by the levels of mitochondrial NAD^+^ (mNAD^+^) [22], not on the levels of cytoplasmic NAD^+^, as previously thought. Using cell culture, we also identified the mitochondrial sirtuin SIRT3 as a downstream mediator of this cell survival pathway. In this study, we explore the physiological relevance of this process in a major post-mitotic tissue, the heart, and identify the acetylation of lysine 166 on CypD as a critical target of SIRT3 that dictates the pace of aging and age-related diseases in cardiac tissue.

## RESULTS

### SIRT3 delays opening of the mPTP in aged cardiac muscle

Based on our previous work, we hypothesized that SIRT3 might increase cell survival by regulating a mitochondrial protein that dictates cell viability. We focused on the heart, a post-mitotic tissue where mitochondria are critical for sustained function, making it an ideal organ system for evaluating mitochondrial function and cell survival during aging and cell stress.

To assess the role of SIRT3 in mitochondrial function and cell survival in the heart, mitochondria were isolated from hearts of both wild-type and SIRT3^−/−^ mice at various ages and tested for alterations in the susceptibility of mitochondria to opening of the mPTP, a well-known characteristic of aged tissues [23]. We utilized the Ca^2+^-overload swelling assay, a sensitive assay for mPTP opening. At 3 and 6 months of age, there was no difference between the wild-type and SIRT3^−/−^ samples (Figure 1A, B) but by 16 months of age, the mitochondria isolated from the hearts of the SIRT3^−/−^ mice were significantly more prone to Ca^2+^-induced mitochondrial swelling (Figure 1C). This sensitivity was completely rescued by the mPTP inhibitor CsA, demonstrating that the defect in the aged SIRT3*^−/−^* tissues was likely caused by increased mPTP opening. Thus, SIRT3 suppresses the increase in mPTP formation in the heart during aging.

### CypD is acetylated on K166

To identify potential interactors that might provide clues as to how SIRT3 prevents mPTP opening during aging, we performed immunoprecipitation experiments with SIRT3 and subjected the precipitated material for identification by mass spectrometry. The predominant interactors with SIRT3 were two components of the mPTP, namely ANT and VDAC. Extensive analysis of these proteins, however, failed to identify an acetylated lysine that was a direct target of SIRT3 (data not shown). Although we did not detect CypD as an interactor in these immunoprecipitation experiments, our observation that CsA reversed the SIRT3^−/−^ mitochondrial phenotype (see Figure 1C), strongly indicated that CypD was directly involved.

**Figure 2.**
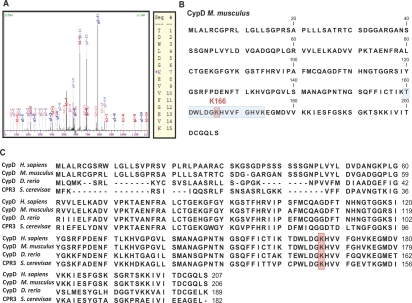
Cyclophilin D is acetylated at K166 (**A**) Representative m/z spectrum of peptide TDWLDG-AcK-HVVFGHVK obtained from mass spectrometry analyses of Flag-purified mouse Cyclophilin D. (**B**) Sequence of Cyclophilin D (*Mus musculus*). Location of the identified acetylation site. (Peptide: blue; acetylated Lysine: red). (**C**) Protein sequence alignment of CypD from *Homo sapiens, Mus musculus, Danio rerio, Saccharomyces cerevisiae*. Lysine 166 is marked in red.

To test this possibility, we first immunoprecipitated CypD and identified lysine 166 (K166) as a site of acetylation (Figure 2A, B). Interestingly this site is highly conserved from yeast to human (Figure 2C). During the course of this study, acetylation of K166 was also described [24] but incorrectly reported as K145 because the authors did not include the mitochondrial targeting sequence. In fact, cyclophilin D (Gene ID: 105675) does not possess a lysine at position 145.

**Figure 3.**
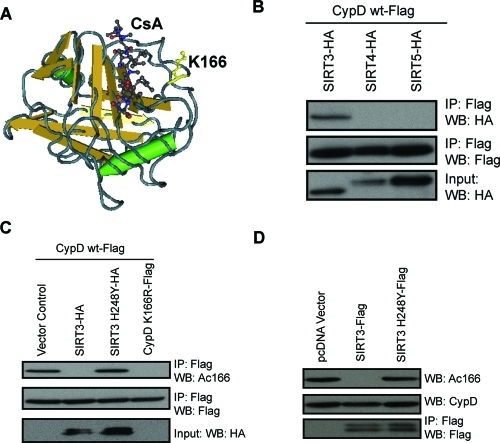
SIRT3 binds to and deacetylates CypD at K166 (**A**) CypD-K166 lies adjacent to the CsA binding pocket (Protein Data Bank, 2Z6W). (**B)**Interaction studies using HA-tagged SIRT3, SIRT4 and SIRT5 and FLAG-tagged CypD assessed by coimmunoprecipitation. (**C**) Specificity of a polyclonal antibody raised against acetylated CypD-K166 confirmed by a lack of Western blot signal for mutant CypD-K166R. SIRT3 and SIRT3-H248Y were co-transfected with FLAG-tagged CypD and the level of acetylation at CypD-K166 was assessed. (**D**) Vectors for FLAG-tagged SIRT3 and SIRT3-H248Y transfected in HEK 293T cells were immunoprecipitated, then incubated with purified CypD in the presence of the SIRT3 co-substrate NAD^+^.

### SIRT3 interacts and deacetylates CypD *in vitro* and *in vivo*

Interestingly, by analysis of the CypD structure [25], we found that CypD-K166 lies adjacent to the CsA binding pocket of CypD (Figure 3A), suggesting that acetylation of K166 on CypD might regulate the mPTP. By co-immunoprecipitation, SIRT3, but not the other mitochondrial sirtuins SIRT4 or SIRT5, interacted with CypD (Figure 3B). To monitor the acetylation status of CypD in cell culture, we generated a polyclonal antibody against the acetylated form of K166 (Ac-K166) using an acetylated peptide (Figure 3C). This antibody did not recognize the mutant CypD-K166R, thus demonstrating its specificity. Coexpression of CypD and SIRT3 resulted in deacetylation of CypD-K166 but not when a catalytic mutant of SIRT3 (H248Y) was expressed (Figure 3C). To test whether SIRT3 can directly deacetylate CypD, we purified both wild-type SIRT3 and SIRT3-H248Y from 293T cells and incubated them with purified CypD in the presence of NAD^+^. Wild-type SIRT3 but not a catalytically inactive SIRT3 mutant (SIRT3-H248Y) deacetylated SIRT3, demonstrating that SIRT3 can directly deacetylate CypD-K166 (Figure 3D).

### SIRT3 knockout mice are hypersensitive to heart stress

Numerous genetic studies implicate formation of the mPTP in the sensitivity of cardiac tissue to stresses including ischemia-reperfusion injury, increased pressure load, and cardiac changes during aging [10, 26]. For example, mice lacking cyclophilin D show reduced infarct size after coronary artery ligation and reperfusion and are relatively resistant to cardiac fibrosis and left ventricle enlargement [26].

**Figure 4.**
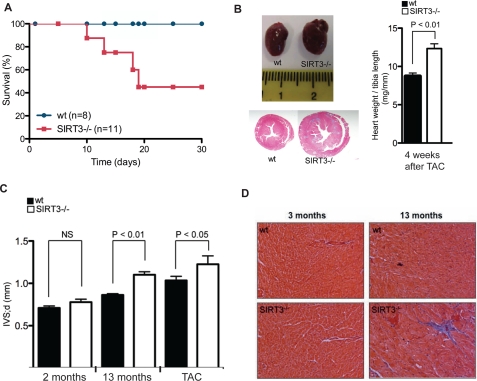
Age- and stress-dependent development of cardiac hypertrophy in SIRT3 ^−/−^ mice. (**A**) Kaplan-Meier survival plot of 3 months old wt and SIRT3^−/−^ mice after the Transverse Aortic Constriction (TAC) surgery. wt (n=8), SIRT3^−/−^ (n=11). p < 0.05. (**B**) Representative image and Masson's trichrome staining of hearts from wt and SIRT3^−/−^ mice 4 weeks after the TAC surgery. Heart weight tibia length ratio of wt and SIRT3^−/−^ mice 30 days after the TAC surgery wt (n=8), SIRT3^−/−^ (n=4) mice. (**C**) Interventricular septal thickness at diastole (Ivs;d) of 2 and 13 month old wt and SIRT3^−/−^ mice after transverse aortic constriction (TAC) for 4 weeks. (**D**) Representative images of Masson's trichrome staining of transverse sections of heart from 13 month old wt and SIRT3^−/−^ mice. Collagen deposits (fibrosis) stain blue. n=4 mice per group and age (magnification, 20x). **p<0.01. *** p < 0.001. All error bars represent s.e.m.

To test whether the interaction between SIRT3 and CypD has physiological consequences for the heart, we subjected SIRT3^−/−^mice to cardiac stress induced by transaortic constriction (TAC), a procedure that increases cardiac afterload and promotes cardiac hypertrophy. Wild type and SIRT3^−/−^male littermates at 3 months of age (n = 8 and 11 respectively) were subjected to a TAC procedure and monitored for 30 days. Half of the SIRT3^−/−^ mice died in the first 20 days following the TAC procedure, whereas none of the wild-type mice died (Figure 4A). In the SIRT3^−/−^ mice that survived, there was a high incidence of cardiac hypertrophy, based on the size of the hearts (Figure 4B) and the size of the left ventricle as measured by echocardiography (Figure 4C).

### SIRT3 delays age-related changes in the heart

To test the function of SIRT3 in the aging heart, we examined the hearts of SIRT3^−/−^mice at multiple time points during their lifespan. Two of the hallmarks of the aging heart are cardiac hypertrophy and fibrosis. We observed an age-dependent increase in left ventricular thickness in SIRT3^−/−^ mice from ages 2 to 13 months, which was comparable to the hypertrophy we observed in the 3-month-old wild-type mice after transverse aortic constriction (TAC). We also observed a greater age-dependent increase in both the left ventricular thickness (Figure 4C) and the formation of cardiac fibrosis (Figure 4D) in hearts from SIRT3^−/−^mice compared to the wild-type controls between ages 3 to 13 months, demonstrating that SIRT3 delays age-related changes in the heart.

## DISCUSSION

We have identified a role for SIRT3 in cell protection and mitochondrial function in heart tissue during induced stress and during aging. Our data show that SIRT3 controls the mPTP and that a loss of SIRT3 activity leads to increased activation of the mPTP in response to Ca^2+^ increases, cardiac stress, and during aging, all resulting in a decline in cardiac function. In this study we focused on the heart, an energy-demanding post-mitotic tissue but we do not rule out the possibility that speculate that this process may affect other energy-demanding organs, a possibility we are currently testing.

**Figure 5.**
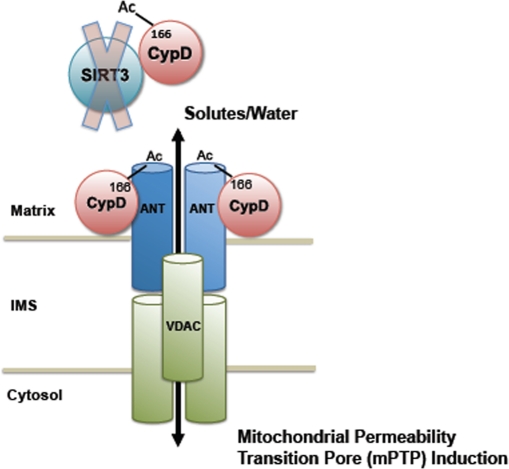
Regulation of the mPTP by SIRT3 In normal cardiac tissue, SIRT3 targets CypD, maintaining it in a deacetylated state, thus preventing opening of the mPTP during aging and induced cardiac stress. In SIRT3^−/−^ mice, however, CypD is hyperacetylated, resulting in increased induction of the mPTP. A decline in SIRT3 activity over time explains the increase in mitochondrial permeability transition and the decline in cardiac function with age. IMS = inner membrane space.

Mitochondrial NAD^+^levels [22] and SIRT3 levels [20, 27] are both known to increase in response to exercise, fasting, and dietary restriction [28]. The ability of SIRT3 to suppress excessive mPTP formation may explain some of health benefits of diet and exercise on cardiac function in middle-aged and elderly people [29]. Conversely, a decline in mNAD^+^ levels during aging or in response to obesity, leading to lower SIRT3 activity, could explain the decline in heart function during aging and why obesity accelerates this decline.

During the course of this study, Sundaresan et al. [30] reported that young SIRT3^−/−^ mice are hypersensitive to agonist-mediated cardiac hypertrophy, which is in agreement with our findings by inducing hypertrophy by the TAC procedure. Their hypothesis to explain the phenotype was that SIRT3 suppresses ROS by activating FOXO-mediated transcription of a manganese superoxide dismutase and catalase. Our results shed light on a more direct and upstream cause of the knockout phenotype that may synergize with a decline in antioxidant defenses. We propose that increased sensitivity to mPTP formation in the absence of SIRT3 promotes mitochondrial leakage, which, in turn, promotes increased ROS production and a reduction in the ability of cells to cope with it. This positive feedback cycle likely damages mitochondria and results in even greater increase of the acetylation state of CypD over time, ultimately leading to mitochondrial dysfunction and destruction.

SIRT3 can also control cell survival by promoting the sequestration of the pro-apoptotic protein Bax to Ku70 within the cytoplasm [31]. Another study showed that SIRT3 stimulates the detachment of hexokinase II from the outer mitochondrial membrane by deacetylating CypD, a step necessary for stimulating oxidative phosphorylation [24]. Together, these findings indicate that by regulating the mPTP, as our data shows, SIRT3 ensures that metabolic and cell survival signals within the mitochondrial matrix are coordinated with those occurring in the cytoplasm.

We propose the following model to explain an age-dependent decline in cardiac function. An initial reduction in SIRT3 activity, perhaps resulting from mitochondrial DNA damage, leads to increases in the susceptibility of mPTP formation. This then reduces the ability of mitochondria to generate NAD^+^[32], leading to a positive feedback cycle that accelerates mitochondrial depolarization and destruction (Figure 5).

Cyclosporin A is a drug used in transplant operations to suppress organ rejection. Its potent suppressive effect on immune function precludes its use to treat chronic diseases, including cardiac hypertrophy and heart failure. This work indicates that a potentially fruitful approach would be to activate SIRT3 directly using small molecules. Small molecules that directly activate SIRT1 have been reported [33, 34], but none yet for SIRT3. It is our hope that this work further stimulates research aimed at finding SIRT3 activators. Such molecules may allow us to delay aging and effectively treat a wide range of age-related diseases.

## METHODS

### Mouse strains and genotyping.

Studies used wild-type and SIRT3^−/−^ 129Sv as described [19]. All animal studies were performed according to IACUC-approved protocols. Mice were genotyped by standard PCR methods as described [19].

### Antibodies and histology.

Antibodies used were specific for SIRT3 (Cell Signaling and [22]), CypD (Mitosciences), Flag (Sigma), HA epitope (Sigma). A rabbit polyclonal antibody was raised against a peptide of CypD, KTDWLDG-AcK-HVVFGH (YenZym Antibodies). Heart sections were stained using Masson Trichrome (IMEB, San Marcos, California).

### Western blotting.

Samples were separated on Tris-HCl 4 - 20% Criterion (BioRad) gels and transferred to nitrocellulose membrane (BioRad). Immunoblots were developed with enhanced chemiluminescence (Denville).

### Plasmid construction and mutagenesis.

All expression constructs were generated by using PCR-based standard cloning strategies, and all expression constructs were verified by DNA sequencing. The mouse CypD coding sequence was PCR-amplified from mouse full-length Mammalian Gene Collection cDNA obtained through Open Biosystems (www.openbiosystems.com) and cloned into the pcDNA3.1+ (Invitrogen)-derived vectors pcDNA^Flag^ to yield CypD with a C-terminal Flag. Site-directed mutagenesis for CypD constructs was performed with QuikChange site-directed mutagenesis kit (Stratagene, La Jolla, CA) as recommended by the manufacturer. pcDNA-hSIRT3-Flag and pcDNA-hSIRT3H248Y-Flag constructs were described previously [19].

### CypD structure and modeling.

The crystal structure of human CypD in complex with its inhibitor Cyclosporin A (PDB ID: 2Z6W) [25] was used as a template and the location of K166 was analyzed by using a 3D-Software (Cn3D).

### Acetylation Analysis by LC-MS/MS.

Excised gel bands were cut into approximately 1 mm^3^ pieces. The samples were reduced with 1 mM DTT for 30 minutes at 60°C and then alkylated with 5 mM iodoacetamide for 15 min in the dark at room temperature. Gel pieces were then subjected to a modified in-gel trypsin digestion procedure. Gel pieces were washed and dehydrated with acetonitrile for 10 min followed by removal of acetonitrile. Pieces were then completely dried in a speed-vac. Rehydration of the gel pieces was with 50 mM ammonium bicarbonate solution containing 12.5 ng/μl modified sequencing-grade trypsin (Promega, Madison, WI) at 4°C. Samples were then placed in a 37°C room overnight. Peptides were later extracted by removing the ammonium bicarbonate solution, followed by one wash with a solution containing 50% acetonitrile and 5% acetic acid. The extracts were then dried in a speed-vac (~1 hr). The samples were then stored at 4°C until analysis. On the day of analysis the samples were reconstituted in 5 μl of HPLC solvent A (2.5% acetonitrile, 0.1% formic acid). A nano-scale reverse-phase HPLC capillary column was created by packing 5 μm C18 spherical silica beads into a fused silica capillary (100 μm inner diameter x 12 cm length) with a flame-drawn tip. After equilibrating the column each sample was pressure-loaded off-line onto the column. The column was then reattached to the HPLC system. A gradient was formed and peptides were eluted with increasing concentrations of solvent B (97.5% acetonitrile, 0.1% formic acid).

As each peptide was eluted they were subjected to electrospray ionization and then they entered into an LTQ-Orbitrap mass spectrometer (ThermoFinnigan, San Jose, CA). Eluting peptides were detected, isolated, and fragmented to produce a tandem mass spectrum of specific fragment ions for each peptide. Peptide sequences (and hence protein identity) were determined by matching protein or translated nucleotide databases with the acquired fragmentation pattern by the software program, Sequest (ThermoFinnigan, San Jose, CA). The modification of 42.0106 mass units to lysine was included in the database searches to determine acetylated peptides. Each acetylated peptide that was determined by the Sequest program was also manually inspected to ensure confidence [35, 36].

### Transverse Aortic Constriction (TAC) surgery.

Mice were anesthetized with isofluorane (1-2% in oxygen, dose to effect) IH. Mice were intubated using a 22GA Angiocath and put on a small animal ventilator CWE. Ventilation parameters were: Tidal volume 0.25ml, Resp. rate 110 br/min, Insp. Time 0.3 sec. The operative field was shaved, cleaned with 10% Betadine and 70% ETOH. A median sternotomy was performed with hemostasis achieved using the Gemini microcautery system. The ascending aorta was dissected from the surrounding connective tissue and a absorbable 6-0 PDS II coated suture by Ethicon without needle was placed around the transverse aorta. The suture was tied down against a blunted 27G needle and the needle was removed to create a chronic model of hypertrophy/heart failure. The chest cavity was closed by continuous absorbable 6-0 PDS II coated suture by Ethicon and 9 mm wound clips for the skin. The animal was put on a warm pad for faster recovery. The mouse was then disconnected from the ventilator and extubated as soon as good spontaneous respirations have resumed. Dehydration was prevented by administration of 1-2 ml/100g body wt. of warm 0.9% saline subcutaneously at the end of the procedure. The surgical procedure was followed by administration of an analgesic (Buprenorphine 0.05 mg/kg subcutaneously (2x a day for first 48h post operatively, minimum 4 doses). A medical record documented the following information at least once a day for at least 48 hours after the procedure: general status, appetite, pain level and quality of incision. Cardiac hypertrophy in mice subjected to TAC was assessed by Mouse echocardiography.

### Mouse echocardiography.

Mice were anesthetized under isoflurane vaporizer (VetEquip) and the paws were secured to the ECG leads on Vevo Mouse Handling Table (VisualSonics Inc.). Body temperature were maintained at 37°C with heating. Chest hair was removed with Nair cream and ultrasound transmission gel was applied. Echocardiography was conducted using a Vevo 770 High-Resolution In Vivo Micro-Imaging System and RMV 707B scanhead (VisualSonics Inc.) with heart rate of 500-550 bpm. M-mode imaging was obtained with parasternal short axis view. Three consecutive cardiac cycles will be measured and averaged according to the Recommendations for a Standard Report for Adult Transthoracic Echocardiography of the American Society of Echocardiography. After the last echocardiography assessment the mice was euthanized by CO2 asphyxiation, followed by cervical dyslocation. Histopathological and proteomic analysis of the hearts of euthanized mice will be performed to determine heart to tibia length ratios. All animals were monitored daily for visual signs of tumors, dehydration, distress or other signs of illnesses.

### Cell culture and transfections.

293T cells were maintained in DMEM supplemented with 10% fetal bovine serum, 2 mM L-glutamine, 100 U/ml penicillin, and 100 μg/ml streptomycin. Cells were transfected using Fugene 6 (Roche) and mitochondria were isolated 48 hr after transfection.

### Isolation of mitochondria.

Mitochondria from mouse hearts were isolated as previously described with slight modifications [37, 38]. Hearts were homogenized in isolation buffer (250 mM Sucrose, 1 mM EGTA, 5 mM HEPES, pH 7.3) containing 1mg of subtilisin A (Sigma) per g of tissue and centrifuged at 2500 rpm for 5 min at 4°C. The pellet was discarded and the supernatant was centrifuged again at 10000 rpm 10 min. The pellet was resuspended in isolation buffer and centrifuged again at 10000 rpm for 10 min. This step was repeated once more and the final pellet was resuspended in isolation buffer without EGTA. Protein concentration was determined using a Bradford protein assay (BioRad).

### Mitochondrial swelling assays.

Calcium-triggered mitochondrial swelling assays were performed as previously described [37]. Briefly, 1 mg/ml mg of heart mitochondria were suspended in MPTP buffer (200mM Sucrose, 10 mM HEPES, 5 mM KH_2_PO_4_, 10 μM EGTA, pH 7.3) supplemented with 10 mM succinate and 1.5 mM rotenone. The mitochondrial swelling was triggered by the addition of 600 μM of CaCl_2_ to heart mitochondria in the presence or absence of 1 μM Cyclosporin A. Mitochondrial swelling was monitored by the decrease in light-scattering at 540 nm in a Cary 50 MPR spectrophotometer (Varian) for 12 minutes [39].

### Immunoprecipitation.

Mitochondria were lysed by sonication and resuspended in a low-stringency IP buffer (0.05% NP-40, 50 mM NaCl, 0.5 mM EDTA, 50 mM Tris-HCl, pH 7.4, 10 mM nicotinamide, 1 μM trichostatin A, protease Inhibitor cocktail (Roche)). CypD (Mitosciences) Antibody was used for Immunoprecipitation of endogenous CypD. FLAG-tagged proteins were immunoprecipitated with anti-FLAG M2-agarose affinity gel (Sigma). Immunoprecipitated material was washed four times for 15 min each in low stringency lysis buffer, and immune complexes were resuspended in SDS-PAGE buffer.

### In vitro deacetylation assays.

Immunoprecipitated material was washed in sirtuin deacetylase buffer (SDAC) (50 mM Tris-HCl (pH 9.0), 4 mM MgCl2, 50 mM NaCl, 0.5 mM DTT) buffer. Reactions were incubated for 3 h at 32°C with constant agitation after addition of NAD^+^ (1 mM).

### Statistical analyses.

Results are given as the mean ± s.e.m. Statistical analyses represent a non-parametric Student's t-test, and null hypotheses were rejected at 0.05.
